# Formula for Extempore Gaseous Chalybeate Water

**Published:** 1846-06

**Authors:** 


*Formula for Extempore Gaseous Chalybeate Water.*—Take of crystallized sulphate of iron two drachms, white sugar three drachms, pulverize and divide into twelve powders. Take of bicarbonate of soda two drachms, sugar three drachms, and divide also in twelve powders. One of them is mixed in half a tumbler of water and drunk while effervescing.—*Ranking.*

